# Effect of climatic variables on production and reproduction traits of colored broiler breeder poultry

**DOI:** 10.14202/vetworld.2015.472-477

**Published:** 2015-04-12

**Authors:** G. D. Nayak, N. C. Behura, K. K. Sardar, P. K. Mishra

**Affiliations:** 1 Department of Animal Breeding and Genetics, College of Veterinary Science and Animal Husbandry, Orissa University of Agriculture & Technology, Bhubaneswar - 751 003, Odisha, India; 2 Department of Poultry Science, College of Veterinary Science and Animal Husbandry, Orissa University of Agriculture & Technology, Bhubaneswar - 751 003, Odisha, India; 3 Department of Pharmacology & Toxicology, College of Veterinary Science and Animal Husbandry, Orissa University of Agriculture & Technology, Bhubaneswar - 751 003, Odisha, India; 4 Department of Poultry Science, College of Veterinary Science and Animal Husbandry, Orissa University of Agriculture & Technology, Bhubaneswar - 751 003, Odisha, India

**Keywords:** Best linear unbiased estimator, climatic variables, colored breeder, multiple regressions, production, reproduction

## Abstract

**Aim:**

The present study was conducted to investigate the important climatic variables affecting production and reproduction in a broiler breeder flock.

**Materials and Methods:**

The experiment was conducted for a period of 1 year on colored synthetic female line male and female poultry birds. 630 female progeny and 194 male progenies from 69 sires and 552 dams produced in four consecutive hatches at an interval of 10 days were used for the present study. Each of the seven, body weight and reproduction traits were regressed with nine environmental variables. Initially, the data were subjected to hatch effect and sire effect corrections through best linear unbiased estimator (BLUE) method and, then, multiple linear regressions of environmental variables on each trait were applied.

**Result:**

The overall regression was significant (p<0.01) in all traits except 20 week age body weight of females. The R^2^ value ranged from 0.12 to 0.90 for the traits. Regression coefficient values (b values) for maximum temperature and minimum temperature were significant (p<0.05) on 5^th^ week age body weight of males. Similarly, evaporation and morning relative humidity (RH) was significant (p<0.05) for 5^th^ week age body weight of females. Almost all b values were significant (p<0.05) for egg production up to 40 week age. The b values representing rainfall, morning RH, afternoon RH, sunshine hours, and rainy days were significant (p<0.05) on bodyweight at 20 week age. All environmental variables except maximum temperature and minimum temperature were significant (p<0.05) on body weight of females at 20 weeks of age. Age at sexual maturity was regressed significantly (p<0.05) with evaporation, afternoon RH whereas, egg shape index was regressed significantly (p<0.05) with a maximum temperature, evaporation and afternoon RH.

**Conclusion:**

The result indicated that various environmental variables play a significant role in production and reproduction of breeder broiler poultry. Controlling these variables in adverse weathers may increase production.

## Introduction

Chicken is the cheapest source of protein available for human consumption, but it cannot tolerate a wide range of climatic variations which affects the production and reproduction. Climate change and animal production always are complementary to each other and its effect on livestock and poultry production is witnessed all over the world [1]. India is more vulnerable due to demographic pressure on natural resources and poor coping up mechanisms. Models are there, which can predict effects of rising temperature, increased climatic variability, and extreme weather events on livestock and poultry production in coming decades. Each individual phenotype is the result of an interaction between the specific genotype and a particular environment. Therefore, genotype × environment interaction is used to describe the situation where, different genotypes (breeds, lines, strains) respond differently to different environments [2]. Temperature, rainfall, solar radiation, atmospheric pressure, etc., were related with fertility. The most potent environmental measures that affect fertility might vary depending on geographical locations. The levels of performance of poultry, does not depend only on inherited capacity but, also to a great extent upon the environment [3].

Poultry production and reproduction are affected by various factors such as, feeding, management, disease control, stock density, housing, climate, sire effect, hatch effect, etc. Research has generated information and techniques to deal with most of these factors except the climate change in order to maximize production. All poultry farmers agree that poultry keeping is an excellent tool in poverty alleviation due to the quick turnover and low investment. Improvement in poultry production implies to create an opportunity for development of the poor section of the society [4]. However, climate change is emerging as a great challenge for poultry industries to sustain the level of production. The worst effects of such climate change are experienced in tropical countries where, a common practice is to house the birds in the open side sheds.

Climate variation is one of the major threats to poultry production. Birds of different breeds/strains and of different age, sex, stage of production, and reproduction respond differently to climatic variations [5]. It is highly desirable that data on such effects in different flocks should be generated and analyzed to develop strategies to deal with adverse effects of climate change.

Available literature on this aspect is scanty to develop a definite strategy and hence, the present study was an attempt to examine and find out important climatic variables affecting production and reproduction in a broiler breeder flock.

## Materials and Methods

### Ethical approval

Ethical approval was not necessary. However, the present research was carried out as per the standard procedures and guidelines of the institution.

### Study period

The study was carried out for a period of 1 year starting from January 2012 to December 2012.

### Study area, population size and collection of data

Flock of colored synthetic female line broiler breeder birds of All India Co-ordinated Research Project on poultry improvement (AICRP on poultry) were maintained at College of Veterinary Science and Animal Husbandry, Orissa University of Agriculture and Technology, Bhubaneswar, Odisha, India. The birds were product of selection for 16 generations for their 5^th^ week, 11^th^ week, and 20^th^ week age body weights. The selection criteria for the progeny selected were individual selection based on 5^th^ week, 11^th^ week, and 20^th^ week age body weights. 630 female progenies and 194 male progenies from 69 sires and 552 dams produced in four consecutive hatches at an interval of 10 days were utilized for this study. The selection process was conducted for 69 sires and 552 dams, and all of them contributed to each hatch in order to produce the next generation progeny, although unequally, hence respective hatch correction was applied for all the individuals. Data on environmental variables were also collected from the observatory of Orissa University of Agriculture and Technology located at Bhubaneswar, Odisha on a daily basis. Feeding, management, vaccination and disease control of the flock under trial were followed adopting a standard procedure. Traits considered for this study were: body weight of male at 5^th^-week of age (g), body weight of male at 20^th^-week of age (g), body weight of female at 5^th^-week of age (g), body weight of female at 20^th^-week of age, female age at sexual maturity (ASM) (days), egg number up to 40 weeks of age, and egg shape index. Egg shape index was equal to 100* (width of egg/length of the egg). The width and length of eggs were calculated through an electronic slide caliper. Environmental variables such as climatic variables considered for this study were: Rainfall (mm), maximum temperature (°C), evaporation (mm), minimum temperature (°C), morning RH (%), afternoon RH (%), bright sunshine (hours), wind velocity (km/h), and rainy days (number). The daily environmental data recorded were totaled from the date of hatch to the date of measurement for production and reproduction traits of individual progeny.

### Statistical analysis

Since the chicks were hatched out in several hatches, all the production and reproduction data were corrected for hatch effect through BLUE estimates [6]. The mathematical model for each trait was Y_ijk_=h_i_+s_j_+e_ijk_ or in matrix notations, Y=Xh+Zs+e

where, Y = observation data of production reproduction traits (n × 1 vector), n=number of observations,

h = p × 1 vector of hatch effect, p = number of levels of hatch effects,

s = q × 1 vector of sire effects, q = number of levels of sire effects,

e = n × 1 vector of random residual effects,

X = design matrix of order n × p, Z = design matrix of order n × q.

Under these assumptions, best linear unbiased estimator (BLUE) equations were reduced to the form,


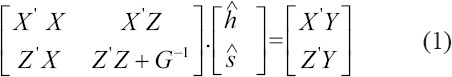


Where, G^−1^ = (1-h^2^)/h^2^; h^2^ is the prior known value of heritability for the trait from literature.

Now, 
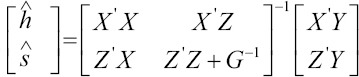


*ĥ*= vector of all hatch effects; *ŝ*= vector of all sire effects.

Estimates for the effects of four hatches were obtained through MS-excel in computer and were subtracted from the production/reproduction data of the progeny belonging to the respective hatch. Then, analysis of variance was conducted using the hatch corrected data in MS-excel following a mathematical model for each trait as described below [7].

Y_ijk_ = µ + s_i_ + d_ij_ + e_ijk;_ dams were nested in sire and

i = 1,2.,s sires; j= 1,2.,d dams per sire; k = 1,2.,n progeny per dam per sire.

Y_ijk_ = observation of k^th^ progeny of the j^th^ dam mated to i^th^ sire.

µ = overall mean; s_i_ = effect of i^th^ sire; d_ij_ = effect of j^th^ dam mated to i^th^ sire; e_ijk_ = the random error associated with Y_ijk_ which is assumed to be normally and independently distributed with mean zero and variance σ^2^.

F-test was carried out to know the significance of sire component. Those traits that were significant (p<0.05) for sire component in the analysis of variance, were subjected to sire effect correction. The individual sire effects calculated through BLUE estimates were taken and were subtracted from the hatch corrected progeny data belonging to respective sires. The effect of climatic variables on each trait was then calculated through multiple linear regressions according using MS-excel data analysis-regression options in the computer [8].

## Results and Discussion

Weekly mean ranges of several climatic variables during different experimental periods are considered because short-term climatic variations affect meat production and quality in livestock and poultry (Table-1) [9]. Mean square values through analysis of variance with nested classification with sire, dam, and error components as sources of variation having the sire component for 20 week age body weight of females and egg shape index were significant (p<0.01), however, all other traits were non-significant for the same (Table-2).

**Table-1:** Weekly values (ranges) of climatic variables at different ages of the poultry flock.

	Age groups				

	0-5 weeks	0-20 weeks	0-25 week	0-40 week	25-40 weeks
Weekly total rainfall (mm)	0-24.6	0-91.7	0-113.6	0-215.3	0-215.3
Mean maximum temperature (°C)	30.2-37.3	30.2-38.4	30.2-38.4	30.2-38.4	30.1-34.1
Mean evaporation (mm)	2.6-6.4	2.6-8.4	2-8.4	1.9-8.4	1.9-5
Mean minimum temperature (°C)	15.7-24.7	15.7-27.2	15.7-27.2	15.7-27.2	16-25.8
Mean morning RH (%)	87-96	84-96	84-96	84-97	84-97
Mean afternoon RH (%)	30-59	30-81	30-89	30-94	35-94
Total bright sunshine (hours)	263.2	929.3	1044.4	1607.93	563.5
Mean wind velocity (km/h)	2.2-13.2	2.2-13.2	2.2-13.2	2.1-13.2	2.1-7
Total rainy days	2	35	60	105	40

**Table-2:** Mean square values of production and reproduction traits of broiler breeder poultry.

	Traits						

	1	2	3	4	5	6	7
Sire	37315.10 (64)	12561.28 (69)	177.43 (68)	191387.20 (66)	479.31 (69)	29.56* (67)	124886* (69)
Dam	3252076.76 (83)	9272.66 (264)	255.36 (203)	216402.20 (87)	382.54 (230)	24.56* (148)	110903.90*(233)
Error	16788.33 (10)	23427.41 (74)	203.40 (155)	184547.40 (40)	352.95 (212)	17.72 (92)	79453.38 (209)

Values in parenthesis indicate respective degrees of freedom.

* Significance at P<0.05, Traits: 1=5th week body weight of male (g); 2=5^th^ week body weight of female (g); 3=Egg number up to 40 week of age, 4=20^th^ week body weight (males); 5=Age at sexual maturity of females (days); 6=Egg shape index; 7=20^th^ week body weight (females)

Regression of different traits on environmental factors with coefficients (b values) along with R^2^ values and F ratio for the overall regression are presented in Table-3. The R^2^ value represents percent variation in the trait which could be explained by the linear regression of nine independent environmental variables. It is the multiple coefficient of determination for the collective effect of all of the independent variables (total rainfall, maximum temperature, evaporation, minimum temperature, morning RH, afternoon RH, bright sunshine hours, wind velocity, and rainy days). F ratio is the F value found in the Analysis of Variance for testing overall significance of the regression.

**Table-3:** Multiple linear regressions on production and reproduction traits of environmental variables in broiler breeder poultry.

Coefficients	5^th^ week body weight (males)	5^th^ week body weight (females)	20^th^ week body weight (males)	20^th^ week body weight (females)	Egg number up to 40 week age	Egg shape index	Age at sexual maturity (females)
a	545.06	−122.45	−69.63	173.99	−24.30	−356.05	−471
b_1_	17.19	6.31	4.36*	−0.36*	−0.01*	−0.11	−0.05
b_2_	0.15*	0.11	−0.22	0.10	0.06*	−0.01*	−0.17
b_3_	3.84	0.59*	−0.75	−0.29*	−0.67*	0.02*	2.07*
b_4_	−0.69*	−0.94	0.05	0.17	-0.02	0.02	−0.26
b_5_	−0.22	0.15*	−0.15*	−0.15*	−0.01*	−0.003	0.004
b_6_	−1.14	−0.51	0.15*	−0.07*	0.04*	0.025*	0.10*
b_7_	2.33*	0.29	−0.31*	0.19*	−0.17*	0.132	−0.32
b_8_	3.63	2.40	0.95	1.28*	0.11*	−0.141	−0.18
b_9_	−88.66	−65.21	−22.69*	1.09*	−0.33*	1.69	0.80
R^2^	0.50	0.12	0.29	0.003	0.08	0.30	0.90
SE (regression)	129.04	102.89	386.42	303.06	14.46	4.04	7.58
F (regression)	19.69**	5.34**	8.45**	0.154	4.32**	12.55**	44.43**

* Significant at *P*<0.05;

** Significant at *P*<0.01,

Subscripts of coefficients: 1. Total rainfall (mm); 2. Maximum temperature (°C); 3. Evaporation (mm); 4. Minimum temperature (°C); 5. Morning RH (%); 6. Afternoon RH (%); 7. Bright sunshine hours; 8. Wind velocity (km/h); 9. Rainy days. F (regression) = F-value calculated through ANOVA for the regression, SE=Standard error

Unpredictable day-length pattern, increase in temperature, unexpected rainfall, high RH, excessive wind velocity, increase in sunshine hours is reported to have a detrimental effect on poultry production. It leads to reduced feed intake, reduced egg production, less body weight gain, small egg size, decreased egg weight, fragile egg shell quality, yolkless egg, reduced feed conversion efficiency [10].

### Climate and 5^th^-week body weight

The result of the regression analysis for the effect of the climatic elements on the 5^th^-week body weight shows that R^2^ is 0.56 for males and 0.12 for females. The implication is that about 56% of the variance of the body weight in males and 12% of the variance in females has been explained by the climatic elements. The F-ratio values are 19.69 and 5.34 for males and females, respectively, and were highly significant at 1% level. This shows that there is a strong relationship between climatic variable selected and the 5^th^-week body weight in both the sexes. However, the difference in variation level between males and females may be due to sex linkage, resulting in higher rate of growth in males than those of the females. Testosterone promotes protein anabolism resulting in increased body size in male as compared to the female. The skeleton also responds to testosterone, with bones becoming larger and thicker [11].

Of the nine climatic elements studied, only maximum temperature, minimum temperature, and bright sunshine hours had significant (p<0.05) influence on 5^th^-week body weight of males. The maximum temperature and bright sunshine hours had a positive effect and minimum temperature had a negative effect on this trait. The chicks were hatched out in cold months of January-February and the atmospheric temperature was low during this time. Any temperature below the ideal brooding temperature (32°C) is likely to affect growth and performance of the birds [12]. It is established that the 5^th^-week body weight is considered for initial selection of progenies. As during the period, the minimum environmental temperature ranged from 15.7°C to 24.7°C (Table-1), this could be the reason for the negative relationship between minimum temperature and 5^th^-week body weight. The maximum temperature is positively correlated with bright sunshine hours as with an increase in bright sunshine hours, the maximum temperature is bound to increase. The positive relationship of 5^th^-week body weight with a maximum temperature and bright sunshine hours can be explained on the basis that the chicks needed more heat during this period [13]. The lethal temperature for birds was about 47°C at which birds could not dissipate body temperature to atmosphere. Consequently, panting occurred and birds died. In hot weather, birds minimized heat production by less eating, mating, and more sitting. When air temperature was low, birds increased body heat production by increasing feed consumption and activity. Birds hatched in hot months (April-May) achieved lesser 5^th^ week body weight in the present study which is in close agreement with the earlier finding reporting that the day length influenced the body weight positively at all ages in broiler breeders [14]

In contrast to the above, the morning RH and evaporation had significant (p<0.05) and positive effect on 5^th^-week body weight of females. The morning RH was 87-96% and evaporation was 2.6-6.4 mm during this period (Table-3) which could be due to the fact that, cool climate in February- March was negated with high RH resulting in increased body weight of females.

### Climate and 20^th^-week body weight

The result of the regression analysis on the effect of the climatic elements on the 20^th^-week body weight shows that R^2^ is 0.29 for males and 0.003 for females. The implication is that about 29% of the variance of the body weight in males and 0.3% of the variance in females has been explained by the climatic elements. The F-ratio values are 8.45 and 0.154, respectively, for males and females. However, the F-ratio for males were highly significant (p<0.01) and in females it was non-significant. This shows that there is a strong relationship between climatic variable selected and the 20^th^-week body weight in males only and not in females. Furthermore, the difference in variation level between males and females may be due to sex linkage, resulting in higher rate of growth in males than the females [13]

Of the nine climatic elements studied, only total rainfall and afternoon RH had significant (p<0.05) and positive effect whereas morning RH, bright sunshine hours, and number of rainy days had significant (p<0.05) but negative effect on 20^th^ week body weight of males (Table-1). Up to the period of 20 weeks, which included the summer months (March-June), there was more sunshine hours leading to high environmental temperature (30.2-38.4°C). As this temperature is higher than the comfort zone of the birds, the feed consumption might have been reduced leading to lower rate of growth [10]. Feed intake decreased by 1.5 g a day for every degree centigrade rise in temperature above 30°C and the negative effect of the sun shine hours on the 20^th^-week body weight can be explained [13]. The morning RH is always higher than the afternoon RH (Table-1). The more the number of rainy days, the more is the RH. Humidity more than 75% affect breathing, feed intake, and its utilization. The RH recorded in the present study ranged from 84-96 in the morning which is above 75% [15]. The high morning RH might be attributed to affect the feed consumption and utilization resulting in a negative effect on growth and 20^th^-week body weight.

The positive relationship of 20^th^-week body weight in males with the total rainfall and afternoon RH can be explained on the basis that with more rainfall particularly in summer months, the environmental temperature goes down. Further, as the afternoon RH is always lower than or around the threshold value of 75%, it does not adversely affect the growth. Chickens regardless of age could not withstand high temperature and high humidity. When the surrounding air was moist, it could not absorb much moisture from respiratory tract and birds pant rapidly [13]. Similarly, when high ambient temperature and humidity prevailed, birds might not be able to exchange enough air by panting to remove heat from the body. Ordinarily, sweating is not the method of heat removal in birds as seen in humans. That might be the probable reason that morning RH, bright sunshine hours, and rainy days had a negative impact on 20-week body weight of males. Opposite to this, afternoon RH and rainfall had a positive impact. Rainfall and wind in summer brings relief to heat stressed birds. Birds reared in hot and humid environment with detrimental heat stress were lighter at 9 weeks of age and birds grew faster in cooler environment of tropical climate and also resulted in reduced growth and egg production which supports the present findings [16,17].

### Climate and ASM

**Figure-1:**
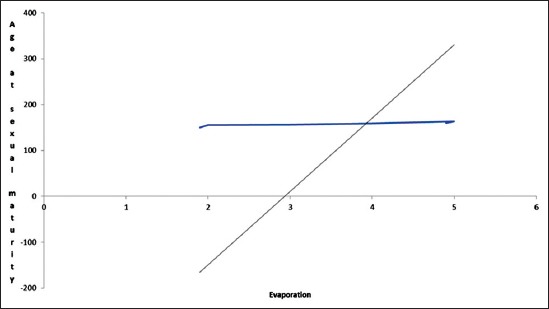
Effect of evaporation of moisture on age at sexual maturity.

The result of the regression analysis on the effect of the climatic elements on the ASM shows that R^2^ is 0.90 (Table-1). It implied that about 90% of the variance of the ASM in females has been explained by the climatic elements. The F-Ratio value was 44.43 and was highly significant at 1% level. This shows that there strong relationship exists between climatic variable selected and ASM. Among all the environmental elements, only evaporation and afternoon RH were regressed positively (p<0.05) with ASM (Figure-1). During 15-20^th^ week of age, the afternoon RH was recorded to be the highest (Table-1). The RH coupled with high environmental temperature might have created an uncomfortable atmosphere delaying sexual maturity. Similar findings have been reported by several workers [14,18]. A statistically significant sire family × temperature interaction for ASM confirmed the present finding [19].

### Climate and 40 weeks egg production

The result of the regression analysis on the effect of the climatic elements on the 40 weeks egg production shows that R^2^ is 0.08 (Table-3). The implication is that about 8% of the variance of the egg production up to 40 weeks has been explained by the climatic elements. The F-ratio value was 4.32 (Table-1) and was highly significant (p<0.01). This showed that a strong relationship exists between climatic variable selected and the 40 weeks egg production.

All environmental coefficients were significant (p<0.05) for egg number up to 40 weeks of age except the evaporation. Egg number was negatively regressed with total rainfall, maximum temperature, minimum temperature, morning RH, and rainy days whereas it was positively regressed with afternoon RH, bright sunshine hours, and wind velocity (Table-3). The egg production started approximately at 25 week of age i.e., in July (rainy season) and continued till October-November (40 week of age), when the climate was relatively cool from scorching heat (Table-1). Hence, wind velocity and afternoon RH were conducive for comfort living of birds leading to more egg production. Moreover, longer bright sunshine hours having photo-stimulation effect on laying hens has resulted in higher egg production [16]. On the contrary, rainfall, morning RH, temperature, and rainy days were more of uncomforting to the breeder hens during that period. Higher rainfall and RH have been reported to increase the disease incidences in the flock leading to low production [20]. Temperature above 80°F was reported to depress egg production, egg size, and shell quality, which is also in agreement with the present findings [21].

### Climate and egg shape index

The result of the regression analysis on the effect of the climatic elements on the egg shape index shows that R^2^ is 0.30 (Table-1). The implication is that about 30% of the variance of the egg shape index has been explained by the climatic elements. The F-Ratio value was 12.55 (Table-1) and was highly significant (p<0.01). This shows that there is a strong relationship exists between climatic variable selected and egg shape index. Egg shape index was regressed negatively (p<0.05) with a maximum temperature indicating that higher the environmental temperature, poorer was the quality of eggshell [20]. Temperature above 21°C decreased feed intake, weight gain, egg production, poor shell quality, and egg size, which supports the present finding [13,22].

## Conclusions

Based on the present finding, it may be concluded that when the chicks are hatched during the months of January-February, environmental temperature, RH, sunshine hours, and number of rainy days are the prominent climatic factors affecting growth, production, and reproduction. The environmental temperature is positively related with growth and negatively related with egg production. Similarly, afternoon RH positively affects growth, ASM, egg number, and egg shape index whereas it negatively influences growth and egg production. Growth and egg production are positively related with Sunshine hours, but negatively influenced by number of rainy days. The findings of the present study can be used to develop models to reduce the influence of potent climatic factors adversely affecting growth, production, and reproduction in breeder poultry hatched during the winter months (January-February). Although the minimum temperature and wind velocity has very less effect on response parameters, but these have been recorded in the present study for further research.

## Authors’ Contributions

GDN and NCB designed the experiment. GDN and PKM collected and analyzed the data. GDN, NCB, and KKS provided technical guidance and participated in the scientific investigation and discussion. GDN and NCB drafted the final manuscript. All authors read and approved the final manuscript.
